# Abnormal visual attention to simple social stimuli in 4-month-old infants at high risk for Autism

**DOI:** 10.1038/s41598-021-95418-4

**Published:** 2021-08-04

**Authors:** Elisa Di Giorgio, Orsola Rosa-Salva, Elisa Frasnelli, Antonio Calcagnì, Marco Lunghi, Maria Luisa Scattoni, Francesca Simion, Giorgio Vallortigara

**Affiliations:** 1 Dipartimento Di Psicologia Dello Sviluppo E Della Socializzazione, Università Degli Studi Di Padova, Via Venezia 8, 35131 Padova, PD Italy; 2 CIMeC, Center for Mind/Brain Sciences, University of Trento, Mattarello, Italy; 3 School of Life Sciences, Lincoln University, Lincoln, UK; 4 Research Coordination and Support Service, Istituto Superiore Di Sanità, Rome, Italy

**Keywords:** Psychology, Human behaviour

## Abstract

Despite an increasing interest in detecting early signs of Autism Spectrum Disorders (ASD), the pathogenesis of the social impairments characterizing ASD is still largely unknown. Atypical visual attention to social stimuli is a potential early marker of the social and communicative deficits of ASD. Some authors hypothesized that such impairments are present from birth, leading to a decline in the subsequent typical functioning of the learning-mechanisms. Others suggested that these early deficits emerge during the transition from subcortically to cortically mediated mechanisms, happening around 2–3 months of age. The present study aimed to provide additional evidence on the origin of the early visual attention disturbance that seems to characterize infants at high risk (HR) for ASD. Four visual preference tasks were used to investigate social attention in 4-month-old HR, compared to low-risk (LR) infants of the same age. Visual attention differences between HR and LR infants emerged only for stimuli depicting a direct eye-gaze, compared to an adverted eye-gaze. Specifically, HR infants showed a significant visual preference for the direct eye-gaze stimulus compared to LR infants, which may indicate a delayed development of the visual preferences normally observed at birth in typically developing infants. No other differences were found between groups. Results are discussed in the light of the hypotheses on the origins of early social visual attention impairments in infants at risk for ASD.

## Introduction

Autism spectrum disorders (ASD) are well-known early onset, neurodevelopmental disorders characterized by qualitative impairments in social communication and interaction, and by restricted, repetitive, and stereotyped behaviors, interests and activities^1^. An increasing interest in timely detection of ASD markers has emerged, mostly driven by the insight that early identification is a fundamental prerequisite for early intervention^2,3^. Nevertheless, the pathogenesis of the social impairments that characterizes ASD is still largely unknown.

Reduced early orienting and attention to social stimuli, such as faces or eye-gaze, but also biological motion^4,5,6,7,8,9,10,11,12^, have been hypothesized to play a crucial role in the development of social impairments found in ASD^13,14^. These early signs are thought to have cascading effects on the typical development of the social brain network^15,16^ see also^17,18,19,20^, restricting the infants’ exposure to typical social interaction and, consequently, interfering with the emergence of critical developmental milestones relevant for adequate social cognition and communication capabilities.

Prospective longitudinal studies of infants at high-risk (HR) for ASD (i.e., siblings of children with ASD, ^21,22,23^) in the first year of life have shed some light on this hypothesis. Although they provided mixed evidence of social attentional impairments at this early age^24,25^, the majority of those studies suggested the presence, at around 6 months of age, of early signs of diminished and/or altered social attention in HR infants who later develop ASD^22,26^. For instance, at this age infants show decreased visual attention to faces and social scenes^27,28^. Likewise, Merin and colleagues^29^ reported atypical visual attention patterns to the eyes during structured face‐to‐face interactions (still-face paradigm) in 6-month-old HR infants. Moreover, a recent study demonstrated that early biases in gaze behavior for face stimuli may be considered as behavioral markers of atypical cerebral lateralization patterns. Six-month-old infants, who received an ASD diagnosis at 3 years, were slower to look at faces presented on the left compared to a group of at low-risk (LR) infants^30^.

Recently, in line with the hypothesis that such impairments may be present well before 6 months^16^, some studies investigated visual attention for social stimuli in HR infants, starting from birth^31,32^.

Di Giorgio and colleagues^32^ reported that 6-day-old HR newborns did not show any visual preference for social stimuli, such as upright schematic faces, direct eye-gaze and biological motion, in contrast to a group of low-risk (LR) newborns (i.e., infants with no hereditary risk of ASD). Results were interpreted as evidence of disruptions from birth in the reflexive processes that bias newborns’ attention towards social stimuli. These reflexive processes are assumed to be mostly subcortically-mediated (but see^33^).

Early anomalies in these mechanisms may then derail the typical specialization of the cortical mechanisms that guide voluntary and experience-dependent attentional control to social stimuli^34^.

An alternative hypothesis, however, postulates that at the basis of ASD social impairments there could be a disturbance or a delay of the shift from reflexive to voluntary cortical-mediated visual attention, typically happening at around 2–3 months of life^35^. The fact that 2-month-old HR infants showed typical visual attention to eye-gaze, which however declined starting from that time-point to 6 months, suggested that some reflexive-like social orienting mechanisms could be intact at birth. On the contrary, it was assumed that the later-developing cortical specialization for these social stimuli fails to emerge in high-risk infants^36^. However, inferring the typical functioning of subcortical mechanisms on the basis of data collected with infants of 2 months could be partially problematic. The reason is that, by a such age, preferential responses to social stimuli are influenced by developing cortical networks and visual experience.

Importantly, a recent longitudinal study demonstrated that HR and LR infants’ visual attention to animate and inanimate stimuli was comparable at 1 week, as well as at 4 and 5 months. Marginal differences between the two groups, with a visual attention decline only for HR infants, were found at 2–3 months of age^31^. These results appear in line with the idea of the integrity of subcortical mechanisms at birth, and their subsequent decline around 2–3 months of age. However, in such study the *NICU Network Neurobehavioral Scale* (NNNS), was administered to infants. The NNNS assesses the neurobehavioral integrity of newborn babies, by investigating their visual attention when presented with the experimenter’s face and voice (i.e., animate stimulus) or with a ball and a rattle (i.e., inanimate stimulus)^37^. Such stimuli greatly differ from those usually employed in well-controlled visual preference paradigms, making it difficult to draw clear conclusions from this study.

To contribute to the understanding of this controversial issue, the present study sought to provide additional evidence on the origin of the early visual attention disturbance that seems to characterize HR risk infants within the first 4–5 months of life. To achieve this goal, well-controlled standardized preferential looking tasks were used to investigate social visual attention in 4-month-old HR and LR infants. The data reported here are a subset of a larger longitudinal study, coordinated by the Italian Network for early detection of Autism Spectrum Disorder (NIDA), in which infant siblings of children with ASD (HR) and of typically developing children (LR) were recruited from birth and followed prospectively up to 36 months. For all infants, in addition to the visual attention tasks described in this paper and conducted at 6–10 days and 16 weeks of age, spontaneous movements and crying data are collected at 10 days and 6, 12, 18 and 24 weeks. Then, from 6 to 36 months, all recruited infants are involved in clinical surveillance. The general aim of the NIDA project is the early detection of behavioral markers of ASD (in the early motor and visual attention development, as well as in the acoustical properties of infants’ crying) to provide timely intervention programs^38^. The results presented here include only data gathered at 4 months of age from tasks investigating visual attention to social and non-social stimuli. From a developmental point of view, to test our hypothesis we had to choose a timepoint that was clearly after the sub-cortical to cortical shift, which occurs at 2–3 months. So, 4 months fulfilled this criterion (while 2–3 months would have been too soon). The same infants, tested at the first time point at birth (between the 6th and the 10th day of life) on the same tasks and throughout the same procedure, did not show any visual preference for either static social stimuli such as an upright face and a direct eye gaze or the biomechanical motion, in contrast to LR newborns^32^.

More specifically, differently from Bradshaw and colleagues^31^, who tested infants with the NNNS, in the current study we tested 4-month-old HR infants with well-controlled visual preference tasks. Our expectation was that, at this age, LR infants should have lost the preference they displayed at birth for the simplified visual social stimuli used in our tasks. This hypothesis appears plausible in light of the study that has shown that at around 5 months of age, infants lose the preference for high-contrast schematic face-like configurations^34^. In effect, these *super*-stimuli might represent optimal configurations for the newborn immature visual system, whereas older infants’ attention might be engaged only by the presentation of more complex and informative social stimuli (see^19^). Likewise, evidence in animal models suggests the presence of transient time windows for the expression of social predispositions. Often, these forms of preferential attention to social stimuli can be observed only in the very first post-natal days (reviewed in^39^). We thus hypothesized that the LR group should not display any preference in our test, contrary to what we observed at birth in the same babies^32^. For the HR group, two different hypotheses could be put forward. On the one hand, we could expect that, similarly to when they were tested at birth^32^, HR infants would show no preference for the social visual stimuli. In this case, no difference should be observed between the performance of the two groups, although this seemingly identical behavior may result from two different developmental pathways. On the other hand, it is also possible to hypothesize that HR infants may show a delayed developmental pathway, rather than a total absence of sensitivity to visual social stimuli. Indeed, the delayed onset of competences is a hallmark characteristic of many developmental disorders^40,41^.

In this case, we could expect to observe the delayed emergence of a preference for the simplified social visual stimuli used in our test, resulting once again in a detectable difference between the LR and HR groups. The aim of the current study was to contrast these two hypotheses.

## Results

**Figure 1:**
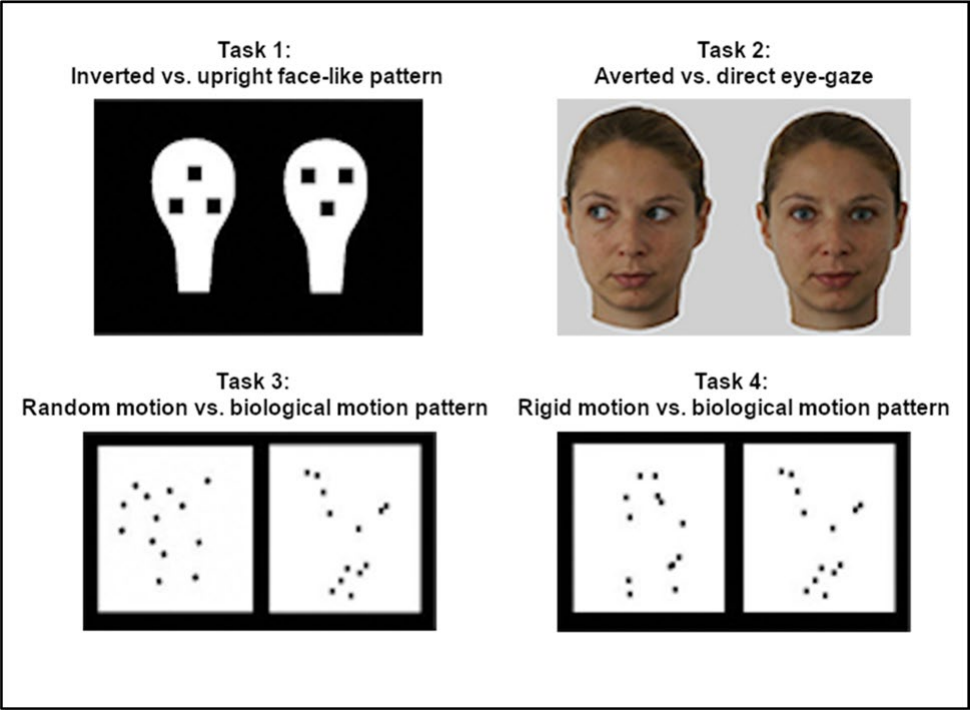
Stimuli used for testing visual preferences. Such stimuli are the same employed in our previous study (Di Giorgio et al.^32^, Scientific Reports) and, as regard with face image, informed consent to publish was obtained from the represented participant.

The apparatus and procedure, as well as the visual stimuli, were the same used in the study conducted by Di Giorgio and colleagues^32^ (Fig. 1). Infants were tested using an infant-control preferential looking technique, and testing took place at home using a mobile lab. The visual behavior was recorded and the videos of infants’ eye movements were coded off-line by an expert coder, unaware of the group to which each infant belonged (HR vs. LR).

Beta linear models were used to assess whether the dependent variables we considered, the *proportion of time fixation* and the *proportion of number fixations* for the non-social stimuli, varied as a function of the variable group (LR and HR) (for details see Methods)^42^. Since the models applied to the proportion of number fixations did not show any statistical significance, only the results for the proportion of time fixation were reported (all the codes and data are available at https://osf.io/m2vax/quickfiles).

**Figure 2:**
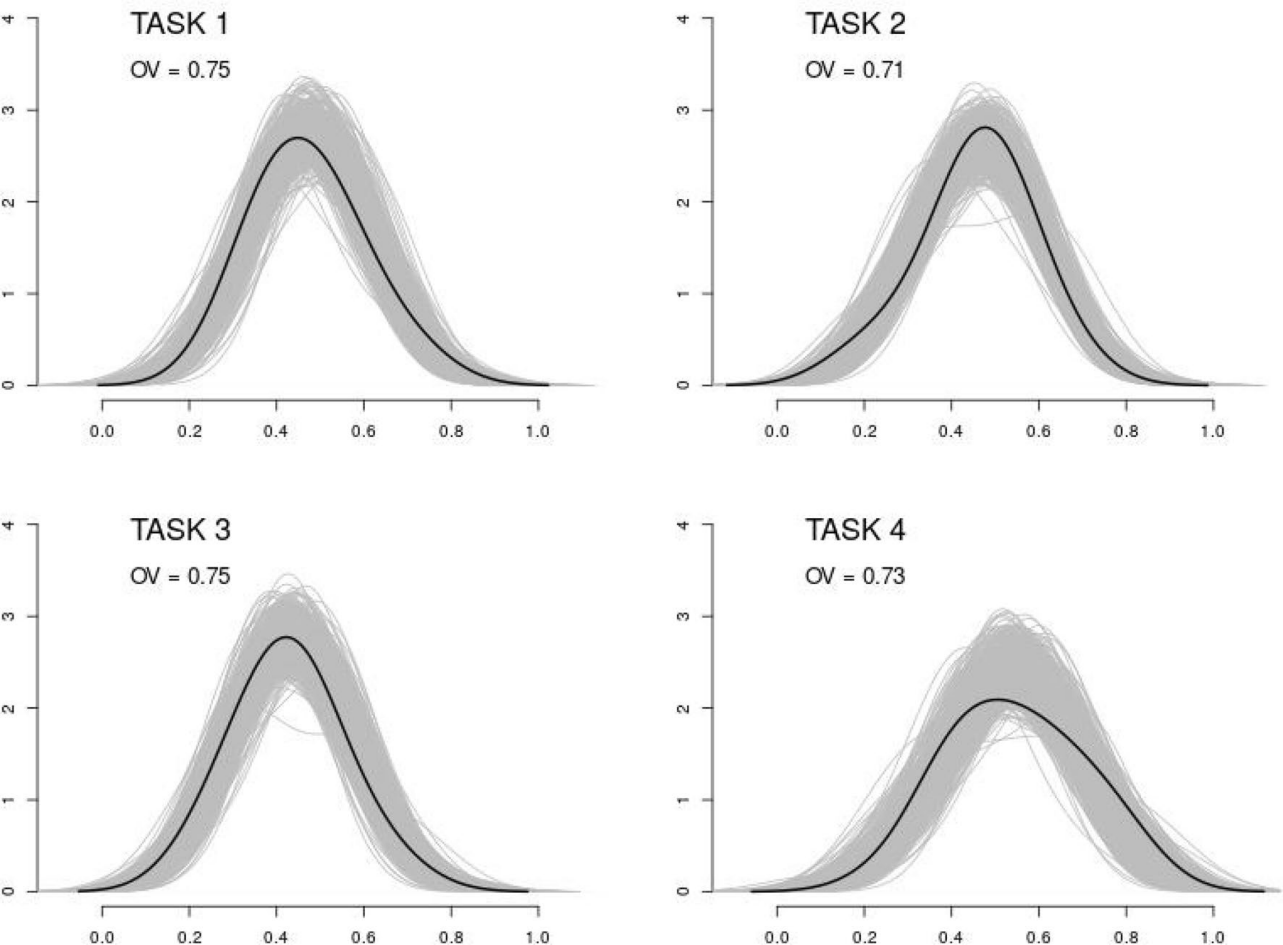
Predicted (in gray) and observed (in black) densities for proportions of time fixation to non-social stimuli. Note that, for all the tasks, densities were estimated using a Gaussian kernel with bandwidth parameter equal to 0.1. Task 1: Inverted *vs.* upright face-like pattern. Task 2: Averted *vs.* direct eye-gaze. Task 3: Random motion *vs.* biological motion pattern. Task 4: Rigid motion *vs.* biological motion pattern.

As for the *proportion of time fixation*, all the models reached the convergence and showed satisfactory fit, as shown by the prediction check densities (in gray color) as opposed to the observed density (in black color) in Fig. 2.

Moreover, the average Overlap index between predicted and observed densities suggested that predicted response proportions highly resembled the observed proportions^43^. Table 1 shows the AIC indices for fixed-dispersion (*M*_*0*_*,* first column) and variable-dispersion (*M*_*1*_*, second* column) beta linear models for each of the four tasks. The models showing lower AIC indices were chosen and subsequently analyzed (see Table 2).

**Table 1:** AIC indices for fixed-dispersion (M_0_) and variable-dispersion (M_1_) beta linear models adapted to each task separately. Note that models with lower AIC are marked with the symbol†.

	M_0_	M_1_
Task1: Inverted *vs.* upright face-like pattern	− 34.13^†^	− 32.39
Task 2: Averted *vs.* direct eye-gaze	− 41.51	− 42.34^†^
Task 3: Random motion *vs.* biological motion pattern	− 35.34^†^	− 33.34
Task 4: Rigid motion *vs.* biological motion pattern	− 23.09	− 24.84^†^

**Table 2:** Estimates and standard errors, *z*-statistic and its *p*-value for the variable dispersion beta linear models. Note that *β*’s denote that parameters for the mean components, *γ’s* denote the parameters for the dispersion components of the model, *ϕ* is the parameter for the overall dispersion parameter. Since the Group variable (HR *vs.* LR) is dichotomic, *β*_*LR*_ is the intercept of the model, therefore it is the baseline.

		Estimate	Std. Error	Z statistic	P-value
Task 1: Inverted *vs.* upright face-like pattern	*β*_ΛΡ_	0.03	0.13	0.2	0.84
	*β*_ΗΡ_	− 0.23	0.17	− 1.3	0.19
	*ϕ*	21.5			
Task 2: Averted *vs.* direct eye-gaze	*β*_ΛΡ_	0.08	0.10	0.86	0.39
	*β*_ΗΡ_	− 0.4	0.15	− 2.73	0.01***
	*γ*_ΛΡ_	3.75	0.44	8.49	0
	*γ*_ΗΡ_	− 0.97	0.54	− 1.79	0.07*
Task 3: Random *vs.* biological motion pattern	*β*_ΛΡ_	− 0.13	0.15	− 0.89	0.37
	*β*_ΗΡ_	− 0.25	0.18	1.38	0.17
	*ϕ*	22.16			
Task 4: Rigid *vs.* biological motion pattern	*β*_ΛΡ_	0.16	0.13	1.25	0.21
	*β*_ΗΡ_	0.04	0.2	0.22	0.82
	*γ*_ΛΡ_	3.31	0.46	7.14	0
	*γ*_ΗΡ_	− 1.17	0.56	− 2.11	0.03**

***p ≤ 0.01, **p ≤ 0.05, *p ≤ 0.1.

In particular, proportions of time fixation in Task 2 (Averted vs. direct eye-gaze) were better modeled by a fixed-dispersion beta model, with participants in HR group showing a significant lower degree of proportions of time fixation for the averted-gaze stimulus, i.e., a higher proportion of time fixation for the direct-gaze stimulus compared to the LR ones (β_HR_ = − 0.40, *p* = 0.01). For this task, the model also included a group-based dispersion component, with participants in HR group showing a significant higher degree of dispersion (γ_HR_ = − 0.97, *p* = 0.07) as opposed to participants in LR group. A similar result was obtained also for Task 4 (Rigid motion *vs.* biological motion patter), in which participants in HR group showed a significant higher dispersion (γ_HR_ = − 1.17, *p* =  0.03) compared to the LR group. However, in this task, the data revealed a non-significant effect of the group for the mean component (β_HR_ = 0.04, *p* = 0.82).

In line to what observed in Task 2, both in Task 1 (Inverted *vs.* upright face-like pattern) and in Task 3 (Random motion *vs.* biological motion pattern), HR infants tended to show a lower degree of proportion of time fixation for the non-social stimuli (higher proportions of time fixation for the social stimuli). However, this tendency was not significant for either task (Task 1: β_HR_ = − 0.23, *p* = 0.19; Task 3: β_HR_ = − 0.25, *p* = 0.17).

**Figure 3:**
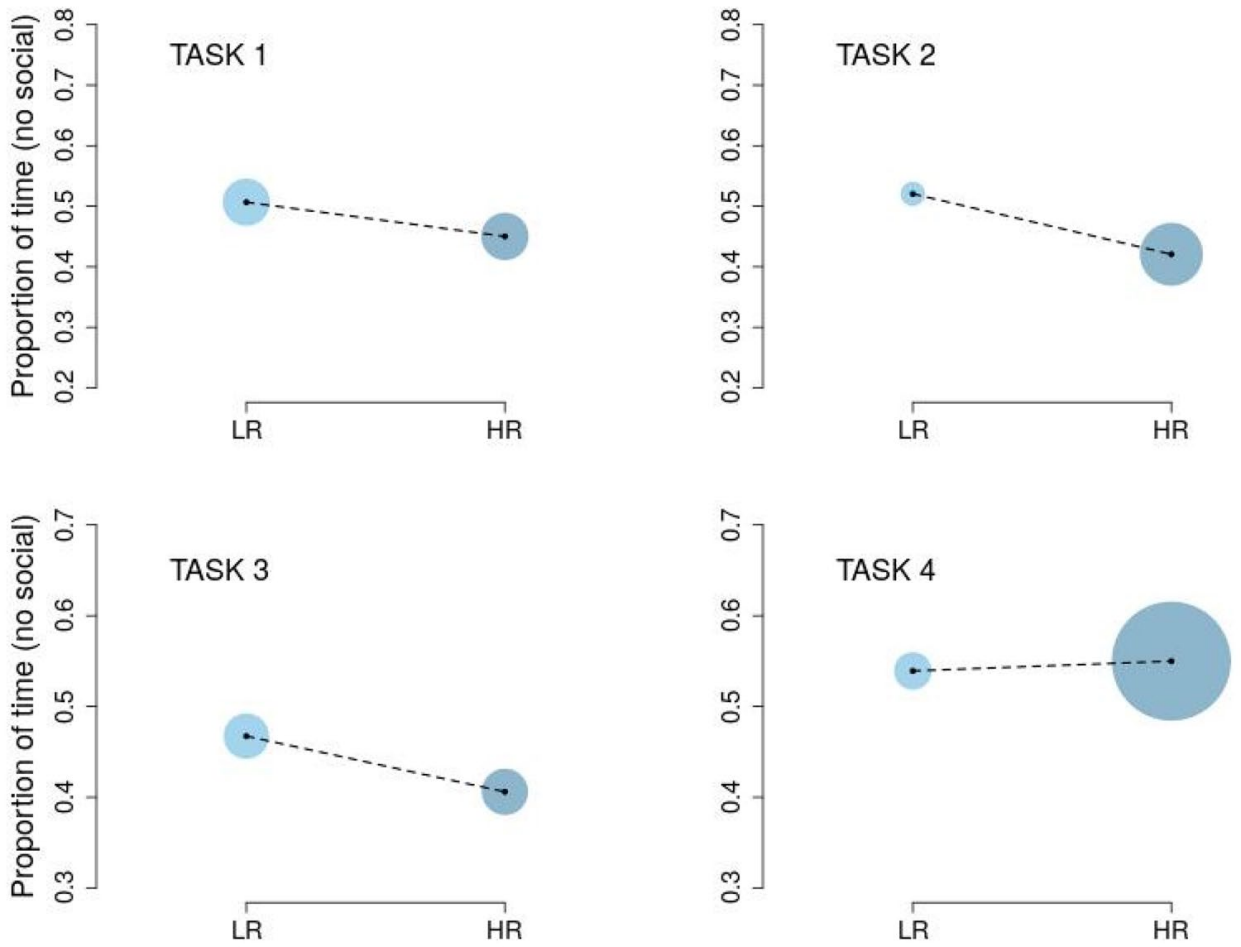
Estimated proportions (small black circles) and dispersion (big colored circles) of time fixation to non-social stimuli for all the tasks and both LR and HR groups. Note that dotted lines between mean points are drawn to highlight the increasing/decreasing levels of both groups and do not indicate any linear trend. Task 1: Inverted *vs.* upright face-like pattern. Task 2: Averted *vs.* direct eye-gaze. Task 3: Random motion *vs.* biological motion pattern. Task 4: Rigid motion *vs.* biological motion pattern.

Figure 3 depicts both the estimated proportions of time fixation to non-social stimuli (black dots) and the associated dispersion components (colored disks). Overall, the results suggested (i) the effect of group for both mean and dispersion components of the response variable in Task 2 and (ii) the effect of group in modulating the dispersion component of the model in Task 4.

**Figure 4:**
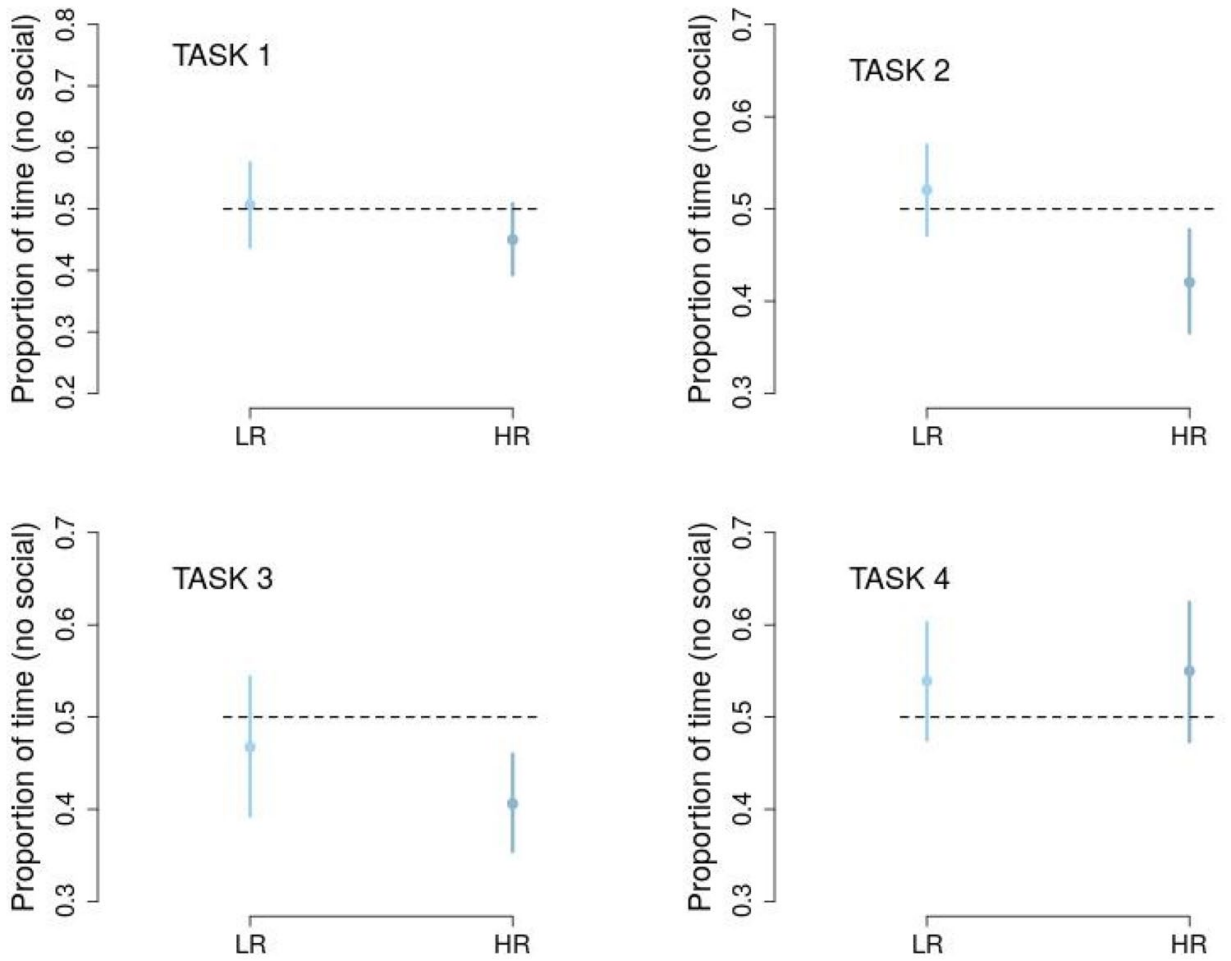
Mean proportion of time fixation and the confidence intervals in each of the four tasks. Mean proportions were calculated on the basis of the beta coefficients of the four linear models (Table 2). The null hypothesis (H_0_ = mean proportions 0.5) is rejected if the confidence interval does not overlap with the chance level (dashed lines). Task 1: Inverted *vs.* upright face-like pattern. Task 2: Averted *vs.* direct eye-gaze. Task 3: Random motion *vs.* biological motion pattern. Task 4: Rigid motion *vs.* biological motion pattern.

Finally, HR and LR infants’ visual preference towards the non-social stimuli in each of the four tasks was investigated. Each value was compared to the chance level of 0.5. HR infants showed a significant visual preference towards the direct eye-gaze (Task 2) (i.e., below the chance level of 0.5 in Fig. 4) and the biological motion when compared with the random motion stimulus (Task 3). No other significant results were obtained.

### Averted vs. direct eye-gaze: a comparison between newborns and 4-month-old infants

**Figure 5:**
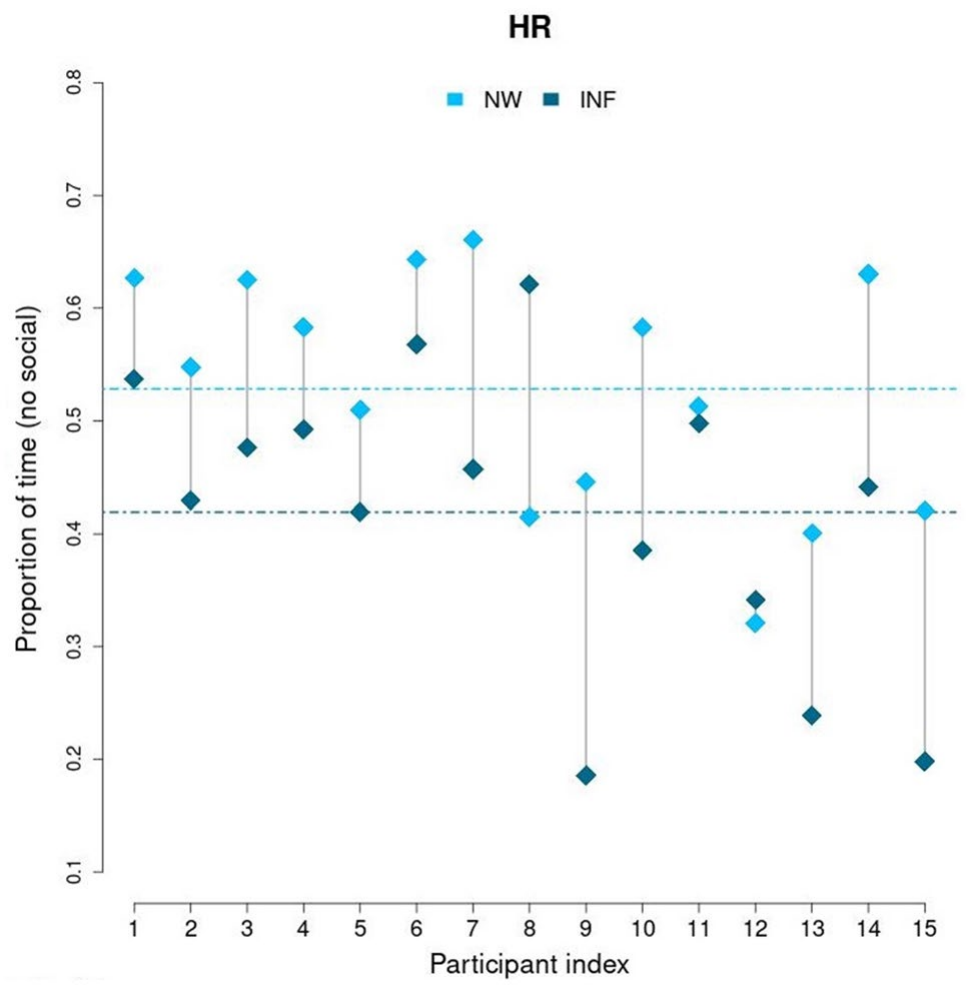
Observed proportion of time fixation in Task 2, Averted vs. direct eye-gaze, for individuals measured at birth (NW = Newborns, light blue color) and at 4 months (INF = Infants, dark blue color). Note that dotted colored lines represent observed means for both time points.

A statistically significant difference between HR and LR was obtained only in Task 2 (adverted vs. directed gaze), where HR infants, in contrast to LR, showed a visual preference for the social direct eye-gaze stimulus. As one of the proposed hypotheses is that HR infants may show a delayed emergence of a visual preference for social stimuli, here we reported how visual behavior changed in Task 2 in individual HR infants (data are reported only for infants who provided analyzable data in each of the two time-points, at birth and at 4 months). As depicted in Fig. 5, it appears that most infants who showed a visual preference for the direct eye-gaze stimulus at 4 months (M_*direct eye-gaze*_= 0.581, SD = 0.130), did not show any visual preference or looked longer at such stimulus at birth (M_*direct eye-gaze*_= 0.472, SD = 0.106).

Indeed, all but one infant, showed an increased preference for the direct gaze stimulus in the second test compared to the first test, revealing a remarkable within-group consistency. This allows us to exclude that the change in the direction of the preference observed at 4 months of age for HR infants, compared to newborns, could be due to a shift occurring only in a minority of individuals, whose extreme behaviour may be driving the results of the whole group. This seems to confirm that the visual behavior observed in HR infants at 4 months could be the result of a delayed emergence of a preference for the simplified social visual stimuli.

## Discussion

The present study, that is part of an ongoing prospective investigation about early behavioral signs of ASD in the first 36 months of life, aimed at investigating social visual attention in 4-month-old infants at HR and LR for ASD.

Visual attention differences between HR and LR infants were found for stimuli depicting a direct eye-gaze, compared to an adverted eye-gaze (Task 2). Specifically, HR infants showed a visual preference for the direct eye-gaze stimulus, in contrast to LR infants, which did not appear to prefer any of the two stimuli.

As for the LR, their lack of preference for the social stimuli is in line with our original hypothesis that these over-simplified static configurations would not be attractive anymore for this age group^34^. Of course, this explanation applies to all task used in the present study and we cannot rule out that it also applies to the direct eye-gaze stimuli. However, for this specific task, the lack of preference may also reflect the earlier stages of the emergence of joint attention mechanisms that would attract typically developing infants towards adverted gazes (see below). Based on the currently available data, we cannot disentangle these two alternative explanations.

As for the HR, at first glance, the emergence of a preference for the direct gaze stimulus may seem quite surprising, in light of the literature describing diminished attention to others’ eyes as an early sign of ASD^36,44^. However, this result may be interpreted considering the typical development trajectory of joint attention – the ability to purposefully coordinate one’s attention to that of another person. Reading social cues conveyed by others’ gaze direction (i.e., averted eye-gaze) to pay attention to an object of interest within the environment represents an early building block for the development of joint attention. This capability starts to emerge in typical developing infants as young as 4 months^45,46,47^. Thus, in line with this interpretation, LR infants at this age are expected to have lost their preference for the direct eye-gaze, due to their emerging interest in the averted-gaze stimulus as a prerequisite for gaze-following behavior (and in line with the idea of a general loss of interest for the oversimplified versions of social stimuli that are attractive at birth). What we observe in HR infants may thus represent the delayed emergence of a visual preference (which they lacked at birth, 32), in line with the second hypothesis that we formulated prior to this study. In turn, this may delay and/or compromise the development of joint attention abilities, potentially contributing to the social attention deficits observed in ASD^48^.

Notably, similar, although not significant, trends for a higher visual preference for the social stimuli in the HR than in the LR group were observed also for Task 1 (comparing schematic face- and non-face-like stimuli) and Task 3 (semi-rigid biological motion vs. random motion). For Task 3, this trend is also supported by the fact that, the HR group (but not the LR one) showed a significant preference for the biological motion stimulus, akin to what observed in Task 2 for the direct gaze (even though this should be interpreted with caution, in the absence of a significant difference between the two groups).

Moreover, the group of HR infants showed also a significantly higher variability than LR infants in their level of preference for stimuli depicting direct eye-gaze (Task 2) and for stimuli depicting biological semi-rigid motion (compared to rigid motion, Task 4). This latter result is particularly intriguing, since it suggests that, even when measures of absolute preference levels do not reveal differences between HR and LR infants, the behavior of babies at higher familiar risk for ASD is more variable. This result could be interpreted considering the higher rate of variability and heterogeneity that characterizes ASD and, even more, the Broader Autism Phenotype (BAP^49^). A greater variability could correspond to a greater susceptibility to risk derailment from the typical visual attention developmental trajectory. Future prospective longitudinal studies with HR infants could further investigate this issue, aiming to confirm the presence of higher variability of social attention in wider samples of HR infants and correlating these measures with other developmental outcomes.

In the present study, LR infants show a lack of visual preference towards social stimuli. This result is in line with a previous study that failed to find a visual preference for social stimuli (i.e., a schematic face-like) in 19-week-old infants^34^. As we mentioned before, while the stimuli presented were adequate to elicit visual preference in LR newborns^11,32,50,51,52^, the same schematic, high-contrast and silent images could be no longer attractive for infants of 4 months of age. It is plausible, therefore, that more engaging stimuli, as well as tasks, could have led to different results. Future studies should specifically investigate which stimuli and paradigms are best suited to elicit visual preferences in typically developing infants at different timepoints.

Overall, except for Task 2, the present study seems to suggest that differences in the visual attention to social and non-social stimuli present at birth^32^, are no longer observable at 4 months in the same LR and HR infants. While non-significant results need to be interpreted with caution, especially due to the small sample size of the current work, this may be in line with studies that found no differences in social attention in HR and LR infants around 4 and 5 months of age^25,31^. The present data appears to be partially in line with what has been observed by Bradshaw et al.^31^, who found no difference between HR and LR infants at 5 months. However, their study showed that the HR and LR groups did not differ at birth but differed at 2–3 months of age, suggesting that impairments were not present at the inborn predisposition level, but in the shift between subcortical and cortical mechanisms that occurs between 2 and 3 months of life. On the contrary, the differences in social predispositions we previously observed at birth^32^ between the same HR and LR infants of this study, suggested an early disruption of subcortical orienting mechanisms, which seems to not support the subsequent specialization of the cortical areas that guides voluntarily attention to social stimuli.

To confirm the hypothesis proposed in the present study on the developmental time course of social and non-social visual attention in HR and its mechanisms, we will also need to correlate the of results the present study with the results from the clinical tests that all participants will be asked to perform at 36 months. Indeed, to better characterize earlier attentional trajectories as precocial behavioral markers of ASD, it will be necessary to compare developmental trajectories of visual attention in HR infants who received a diagnosis of ASD with those who have not received it, which will be the objective of our future studies.

In conclusion, in attempting to give more accurate answers about how early anomalies of visual attention to social stimuli have a cascading effect on later emerging social capabilities, it is mandatory to monitor the development trajectory of visual attention for such stimuli in HR infants.

## Methods

### Recruitment and participants

Pregnant mothers of HR or LR infants were enrolled from various regions throughout Italy. Informed consent was obtained from the pregnant mothers included in our sample.

Recruitment, informed consent and ethical approval were made available through the NIDA Network. All methods were carried out in accordance with guidelines approved and all the study procedures were approved by the Ethics Committee of the Istituto Superiore di Sanità in Rome (Italy) for this project (approval code Istituto Superiore di Sanità CE/11/308).

All the babies included in the sample were healthy and full-term and were tested at home only if awake and after the parents had provided informed consent. For both HR and LR groups, the exclusion criteria included gestational age < 37 weeks, low birth weight (< 2500 g), Apgar score < 7 at the 5th minute, presence of other medical, genetic or neurological conditions and, clinically, pre- or perinatal complications.

Participants included 21 infants (7 males, M_age_ = 17.5 weeks, SD_age_ = 4.2) in the HR group and 19 infants (9 males, M_age_ = 16.3 weeks, SD_age_ = 3.8) in the LR group (which had no familial history of ASDs).

All infants in the HR group met the screening criteria adopted by the guidelines proposed by the NIDA network, by having an older sibling who had received a clinical diagnosis of an autism spectrum disorder via diagnostic evaluation by a licensed clinical psychologist or a medical doctor. Five babies in the HR group were excluded from analyses because they started crying during testing (n = 3) or because other medical and neurological conditions were diagnosed after recruitment, including visual impairment (n = 2). In the LR group, 1 baby was discarded because she started to cry at the beginning of the experimental session.

### Apparatus and procedure

The mobile lab consisted of a computer monitor (Dell Display Flat Panel 27″, refresh rate = 60 Hz, 1240 × 768) where stimuli were presented, a laptop with E-prime 2.0 software^53^ for stimuli presentation only (HP Elitebook 8570P), a high-resolution video camera (JVC TK-C750 U) placed above the monitor to record the eye movements of the infants during stimuli presentation, a mixer (Datavideo HS-600) to assemble the stimuli from the laptop with the output of the video camera, and a video recorder to save the videos of the infants’ eyes and allow off-line coding of the eye movements.

Infants were tested with four different tasks, each comprising two trials. Two visual stimuli were simultaneously presented in each trial and the relative left/right position of each stimulus in the pair was counterbalanced across trials. Since the procedure used was infant-control, each trials ended when the infants stopped looking at the displays for at least 10 s. Importantly, all infants were presented with all four tasks and the order of presentation was randomized between participants.

During the tests, infants sat on an experimenter’s lap at a distance of about 30 cm from the monitor. The camera placed above the monitor recorded the eye movements of the baby to register their looking behavior. At the beginning of each trial, a red disc on a black background was used as attention getter. The disc grew and shrank back continuously (oscillating between 1.8 cm and 2.5 cm diameter) to attract the infants’ gaze to the center of the screen. As soon the infant was looking at the red disc, a second experimenter, monitoring the baby’s attention (by observing a feed of the video of the baby’s eyes presented on the mixer monitor), started the sequence of trials by pressing a key on the keyboard that turned off the red disc and turned on the stimuli.

Videos of infants’ eye movements were coded off-line by an expert coder, unaware of the group to which each infant belonged (HR vs. LR).

In accordance with Cohen’s attentional model^54^, the proportion of time fixation was considered as index of detection mechanism. Such proportions were obtained computing the total length of time for which each infant looked at a given stimulus (the social or the non-social one) divided by the total length of time towards both stimuli in each visual preference task. The orienting mechanism was indexed by the proportion of number fixations, that consists in the sum of fixations (or orienting responses) towards a given stimulus divided by the total sum of fixation towards both stimuli in each visual preference task. As in the previous study with the same stimuli and tasks^32^, we calculated both the proportion indexes in relation to the non-social stimulus of each pair (inverted face-like, adverted eye-gaze, rigid and random motion). Thus, proportions significantly above the chance level (0.5) indicated a higher deployment of visual attention for the non-social stimulus in each of the four tasks (Fig. 4).

### Stimuli

#### Task 1: Inverted face-like vs. upright face-like pattern

The stimuli consisted of static images of two head-shaped two-dimensional white forms, 22 × 15 cm (36° × 27°), with three black squares (2.5 × 2.5 cm, 4.8° × 4.8°).

In the upright face-like pattern, the black squares were arranged in a triangular fashion and placed in the correct locations to represent the eyes and the mouth of a schematic human face. In the inverted face-like pattern, the triangular arrangement of the black squares was rotated by 180° on the vertical axis^50^. The distance between the two stimuli on the screen was 10 cm (19°).

#### Task 2—Averted vs. direct eye-gaze

These stimuli were colored static images of real female faces orienting their gaze to one side (averted eye-gaze) or gaze straight-on to the observer (direct eye-gaze)^51^. The stimuli were 19.5 × 15.5 cm (33° × 27°). The distance between the two stimuli on the screen was 10 cm (19°). To note, a face with direct eye-gaze is considered social because, compared to a face with averted-gaze, this is the optimal stimuli for activating the Conspec mechanism and the subcortical route that underlies face detection^15^.

#### Task 3—Random motion vs. biological motion pattern

The stimuli consisted of two videos (AVI) representing 13 moving black squares on a white background. The biological motion stimulus represented the pattern of motion of a walking hen, whereas in the random motion stimulus the same squares moved randomly on the background (non-biological motion)^11^. The dimensions of the stimuli were 14.5 × 16.5 cm (26° × 29°) and each black square measured 0.6 × 0.6 cm (1° × 1°) on the screen. The distance between the two stimuli on the screen was 9.5 cm (18°).

#### Task 4—Rigid motion vs. biological motion pattern

Also in this visual preference task, stimuli were two videos (AVI). The same biological motion pattern described above (walking hen) was contrasted with a non-biological, rigid motion pattern, created by rotating the first frame of the hen animation around its vertical axis^52^. The dimensions of the stimuli and the distance between them were the same as those of the previous pair.

### Statistical analysis

Beta linear models were employed to assess whether the *proportion of time fixation* and the *proportion of number fixations* for the non-social stimuli varied as a function of the variable group (LR and HR). Unlike other linear models for bounded response variables (e.g., Logistic), beta regressions account for the heterogeneity usually present in proportions by means of two submodels, namely a linear decomposition of the mean (μ = g^−1^(Xβ)) and dispersion (φ = g^−1^(Xγ)) components, respectively^42^. Thus, four beta linear models were defined and adapted to each task separately. For each task, the plausibility of the variable-dispersion beta model was assessed by contrasting this model against the simplest fixed-dispersion beta model (i.e., a model where the dispersion components fixed over each statistical unit). The Akaike information index (AIC) was adopted to evaluate both the fits and the model showing lower AIC was finally chosen. The overall fit of the models was evaluated by means of a visual predictive check analysis. Finally, the significance of the model’s parameters was evaluated using the *z-*statistic. All the analyses were performed using the R framework for statistical analyses. The beta linear models were adapted using marginal maximum likelihood as implemented in the glmmTMB library^55^. Since the models applied to the proportion of number fixations did not show any statistical significance, only the results for the proportion of time fixation were reported.

## Data Availability

All the codes and data are available at https://osf.io/m2vax/quickfiles.
